# Which low- and middle-income countries have midwife-led birthing centres and what are the main characteristics of these centres? A scoping review and scoping survey

**DOI:** 10.1016/j.midw.2023.103717

**Published:** 2023-08

**Authors:** Andrea Nove, Oliva Bazirete, Kirsty Hughes, Sabera Turkmani, Emily Callander, Vanessa Scarf, Mandy Forrester, Shree Mandke, Sally Pairman, Caroline SE Homer

**Affiliations:** a Novametrics Ltd, Duffield, Derbyshire, UK; b University of Rwanda School of Nursing and Midwifery, Kigali, Rwanda; c Burnet Institute Global Women's and Newborn Health Group, Melbourne, Vic, Australia; d Monash University Health Systems Services & Policy Unit, Melbourne, Vic, Australia; e University of Technology Sydney School of Nursing and Midwifery, Sydney, NSW, Australia; f International Confederation of Midwives, The Hague, The Netherlands

**Keywords:** Midwifery, Midwife-led, Childbirth, Model of care

## Abstract

Evidence about the safety and benefits of midwife-led care during childbirth has led to midwife-led settings being recommended for women with uncomplicated pregnancies. However, most of the research on this topic comes from high-income countries. Relatively little is known about the availability and characteristics of midwife-led birthing centres in low- and middle-income countries (LMICs). This study aimed to identify which LMICs have midwife-led birthing centres, and their main characteristics.

The study was conducted in two parts: a scoping review of peer-reviewed and grey literature, and a scoping survey of professional midwives’ associations and United Nations Population Fund country offices. We used nine academic databases and the Google search engine, to locate literature describing birthing centres in LMICs in which midwives or nurse-midwives were the lead care providers. The review included 101 items published between January 2012 and February 2022. The survey consisted of a structured online questionnaire, and responses were received from 77 of the world's 137 low- and middle-income countries.

We found at least one piece of evidence indicating that midwife-led birthing centres existed in 57 low- and middle-income countries. The evidence was relatively strong for 24 of these countries, i.e. there was evidence from at least two of the three types of source (peer-reviewed literature, grey literature, and survey). Only 14 of them featured in the peer-reviewed literature. Low- and lower-middle-income countries were more likely than upper-middle-income countries to have midwife-led birthing centres. The most common type of midwife-led birthing centre was freestanding. Public-sector midwife-led birthing centres were more common in middle-income than in low-income countries. Some were staffed entirely by midwives and some by a multidisciplinary team. We identified challenges to the midwifery philosophy of care and to effective referral systems.

The peer-reviewed literature does not provide a comprehensive picture of the locations and characteristics of midwife-led birthing centres in low- and middle-income countries. Many of our findings echo those from high-income countries, but some appear to be specific to some or all low- and middle-income countries. The study highlights knowledge gaps, including a lack of evidence about the impact and costs of midwife-led birthing centres in low- and middle-income countries.

## Highlights

• Midwife-led birthing centres exist in at least 24 low- and middle-income countries.
• Many of these countries do not feature in the peer-reviewed literature.
• There are some similarities and some differences with high-income countries.
• The impact and costs of midwife-led birthing centres are under-researched.

## Introduction

There is extensive evidence of the safety and benefits of midwives and midwife-led care during pregnancy, childbirth and the postpartum period (Betrán et al., 2018; Nove et al., 2021; Sandall et al., 2016). In some countries, this evidence has led to midwife-led birthing centres (MLBCs) being recommended as the safest birthplace for women with uncomplicated pregnancies (National Institute for Health and Care Excellence, 2017). However, widespread access to midwife-led birthing care seems largely confined to high-income countries (HICs) (Edmonds et al., 2020). The existence and impact of this model of care in low- and middle-income countries (LMICs) is less well researched. These contexts are important given the majority of maternal morbidity and mortality occurs in LMICs, (World Health Organization et al., 2023) hence the potential impact is high.

The International Confederation of Midwives (ICM) led a study which aimed to find out ‘what works’ and ‘why’ in relation to midwife-led birthing centres (MLBCs) in LMICs. This paper reports on the first phase of this study, which aimed to document the LMICs which have MLBCs and the main characteristics of these MLBCs. This analysis complements existing knowledge to yield a more comprehensive understanding of MLBCs throughout the world.

ICM's current working definition of an MLBC is “*a healthcare facility offering birth and sexual and reproductive health care services, using the midwifery model of care. It specializes in care for routine birth, ensures access to emergency care, and is fully integrated within the healthcare system. A midwifery centre is distinguished by its alignment with the midwifery philosophy of care. This human-rights-based, woman-centred approach, is expressed through a home-like shared space that encourages participation of the woman, and her community. The midwifery centre aligns the level of care provided to changing needs, staying alert and responsive, to provide an optimal outcome. The care provided at a midwifery centre is orientated and directed towards the woman's experience*” (Stevens and Alonso, 2020). A broader definition was used for this review so as to be as inclusive as possible: “*a dedicated space offering childbirth care, in which midwives take primary professional responsibility for birthing care*”. Within this overall definition, different types of MLBC exist: freestanding (located on a separate site from a hospital obstetric unit), alongside (located on the same site as the obstetric unit but not within it), and onsite (located within the hospital obstetric unit).

MLBCs share characteristics with midwife-led care (MLC, in which the midwife is the lead health care professional, International Confederation of Midwives 2017b) and midwife-led continuity of care (MLCC, in which a known midwife or small group of midwives supports a client throughout the antenatal, birthing, and postnatal continuum, World Health Organization 2016). The distinguishing characteristics of MLBCs are that they (a) provide birthing care but not necessarily antenatal care (although many also provide antenatal and other elements of care along the continuum), and (b) do not necessarily provide continuity of midwifery care (although some do). Previous reviews have considered MLC and MLCC including in LMICs (Batinelli et al., 2019; Michel-Schuldt et al., 2020b), but to our knowledge only one has focused on MLBCs in LMICs, and that study considered only onsite MLBCs (Long et al., 2016).

Previously, 55 countries were identified by the GoodBirth Midwifery Centre Atlas (Goodbirth.net, 2021) as having “midwifery centres”, of which 33 were LMICs, but no definition of a “midwifery centre” was given. Through its network of professional midwives associations, ICM was aware that not all MLBCs were included in this Atlas, and also that not all of the centres in the Atlas provided birthing care, hence the need for this review.

This study's primary research question was: in which LMICs do MLBCs exist? Its secondary research question was: what are the main characteristics of MLBCs in LMICs in terms of nomenclature, urban/rural location, type (freestanding, alongside, onsite), sector, staffing models, services offered, costs and payment mechanisms and impact? These review questions closely align with established criteria for scoping reviews, i.e. the aims are to: identify the types of available evidence, clarify key concepts/definitions, identify key characteristics or factors related to a concept and/or identify knowledge gaps (Munn et al., 2018).

The decision to restrict the study to LMICs was taken because (a) this topic is already well researched in many HICs and (b) we cannot assume that the existing research can be generalised to all settings. We are therefore using the LMIC classification as a starting point rather than as a way of describing a group of countries (Khan et al., 2022; Lencucha and Neupane, 2022). It is clear from this and other studies that LMICs should not be considered as a homogenous group in relation to the configuration of childbirth care services.

## Methods

The study was undertaken in two parts. Part 1 was a scoping review of the peer-reviewed and grey literature, and Part 2 was a scoping survey of professional midwives’ associations. No protocol was published in advance of this work.

### Part 1: scoping review of peer-reviewed and grey literature

The scoping review was guided by established good practice for such reviews (Arksey and O'Malley, 2005; Daudt et al., 2013). The search strategy was informed by a preliminary PubMed and Google search using the terms “midwife-led unit”, “midwifery unit” and “birth centre”, to help identify relevant search terms and to locate earlier relevant reviews. In this study, both midwives and nurse-midwives were counted as midwives, in recognition of the fact that different countries configure their midwifery workforce in different ways. The search terms were designed to capture literature pertaining to nurse-midwives as well as midwives.

The search for peer-reviewed literature was performed on 24 and 25 February 2022, using nine databases and a variety of search terms (see supplementary file, Table S.1). Inclusion and exclusion criteria are shown in Table 1.

**Table 1:** Inclusion and exclusion criteria for peer-reviewed and grey literature.

Inclusion criteria	Exclusion criteria
Population	
Care provided by midwives or nurse-midwives	Care provided by other health workers such as nurses without formal midwifery training, doctors, associate/auxiliary midwives, community health workers and traditional birth attendants
Care provided in low- and middle-income countries as defined by the World Bank	Care provided in high-income countries as defined by the World Bank
**Intervention/comparison**	
Care where a midwife was the lead professional (whether a single midwife working alone, in a small team of midwives, a caseload model, or within an interdisciplinary team)	Care provided by midwives under the direction of a doctor or other health professional, or by midwives who are the lead professional only by default, i.e. the midwife is the only available professional but there is no obvious commitment to the philosophy of midwife-led care
Care provided in a dedicated (midwife-led) space either within or outside of a health facility*	Care provided in another type of space within a health facility (e.g. a maternity ward or obstetric unit) or outside of a health facility (e.g. at the client's home)
Care includes (but is not necessarily limited to) childbirth	Care does not include childbirth
**Outcome**	
Existence of one or more spaces where midwife-led birthing care is provided	All other outcomes or none
**Study design**	
Item is a research study, report of activities, opinion piece, or conference abstract	Item is a review of the literature
Year of publication was 2012 or later	Year of publication was before 2012
Published in English, French or Spanish	Published in other languages

⁎ We did not exclude facilities if they did not fully meet the ICM working definition of an MLBC, because one of our aims was to identify and describe their characteristics, rather than to assume that the working definition applies in all contexts.

Items identified through the searches were screened by a team of six researchers, using Covidence software (Covidence, 2022). After removal of duplicates, each title and abstract was screened by two researchers and a decision made whether or not to put the item forward for full text review. If the two reviewers disagreed the item was discussed by the wider team and a consensus decision reached. If, during full text review, it became apparent that an item did not meet the inclusion criteria, it was excluded and the reason noted. The team also manually searched the reference lists of relevant, retrieved publications to identify additional items.

During the full-text review, relevant information about each item was recorded in an extraction grid (Table 2). A pilot test of the extraction grid was conducted on the first ten papers, and some adjustments and additions were made before the main stage of data extraction commenced. One researcher extracted the information and recorded it in the grid, then a second researcher read the same paper and checked the information recorded by the first reviewer. Reviewers made informal notes about study quality, but we did not undertake a systematic appraisal of the quality or weight of evidence because this review aimed to provide a descriptive overview of the literature (Pham et al., 2014). Areas of disagreement were discussed by the whole team and resolved by consensus.

**Table 2:** Variables recorded in the extraction grid.

Characteristics of the study	Country/ies
	Number(s) and location(s) of MLBCs mentioned
	Names and definitions used for MLBCs
	Language
	Type of publication
	Year of publication
	Aim(s) of study
	Summary of design/methods
Characteristics of the MLBC(s)	Sector (public, private for profit, private not for profit)
	Type (onsite, alongside, freestanding)
	Births per month
	Model of care (lone midwife, team/caseload midwifery, multidisciplinary team, other)
	Services offered (childbirth care, antenatal care, postnatal care, family planning, other)
Costs and payment mechanisms	Summary of information provided on costs
	Cost comparison made with other model(s) of care?
	Payment mechanism(s) (user fees, insurance, public funds, donor/NGO funds, other)
Measurement	Comparator(s) if any
	Outcome(s) used to measure impact
	Summary of outcome results
Enablers and challenges	Enablers identified
	Challenges identified

Grey literature was obtained in March–April 2022 *via* a Google search and searches of websites of midwifery associations, health ministries, national and international governmental and non-governmental organisations. Search terms included the name of the country combined with each of the following terms: “midwife-led”, “birth centre”, “birthing centre”, “normal birth centre”, “natural birth centre”, “midwifery unit”, or “midwifery clinic”. For French-speaking countries, the search terms included the country name plus “maison de naissance” or “maison d'accouchement”, and for Spanish-speaking countries we searched for the country name plus “casa de parto” or “centro de parto”. There were no restrictions on article type. The main focus was on countries which did not feature in the peer-reviewed literature, but some grey literature was found from the countries in the peer-reviewed literature.

A content and thematic analysis was conducted to identify or quantify the information contained with the extraction grid, and content relating to the review questions.

### Part 2: scoping survey of professional midwives’ associations

According to the World Bank, in 2022 there were 137 LMICs (World Bank, 2022), all of which were eligible for inclusion in the scoping survey. ICM had a member association or a contact in 83 of the 137 (61%), and they were invited by ICM to participate. For 51 of the countries with no ICM contact, the United Nations Population Fund (UNFPA) country office was invited by UNFPA head office to participate. Thus, 134 LMICs were invited to complete an online questionnaire (98% of all LMICs). The three exceptions were: American Samoa, Russia, and Ukraine (American Samoa and Russia because they had neither an ICM member association nor a UNFPA country office, and Ukraine because the ongoing conflict meant that the UNFPA country office was accepting no communication unless related to humanitarian programmes).

A short quantitative questionnaire was developed by the project's technical working group, consisting of experts from ICM, UNFPA and academia. The questionnaire contained 18 questions to establish (a) whether or not MLBCs existed in the country, and if so (b) some information about their characteristics. The questionnaire was developed in English and translated into French and Spanish. The English language version is available in the supplementary file. Respondents were invited to provide their answers online, using the Survey Monkey platform. Invitations to complete the questionnaire were issued by email between 25 and 31 March 2022. Email reminders were sent to non-responding countries between 6 and 14 April 2022. A final reminder was sent to non-responding countries on 26 April 2022. By the closing date of 6 May 2022, responses had been received from 77 countries: a 57% response rate. Details of the responding and non-responding countries can be seen in Table S.2 of the supplementary file.

We compared the survey responses with the findings of the literature review. If the survey response contradicted the evidence from the literature, we contacted the survey respondent to request clarification. As a result, some survey responses were changed and resubmitted (e.g. if the respondent had misunderstood the definition of an MLBC). The final survey responses were analysed descriptively, using frequency counts and contingency tables.

## Results

### Number of items of literature identified *via* the scoping review

**Fig. 1:**
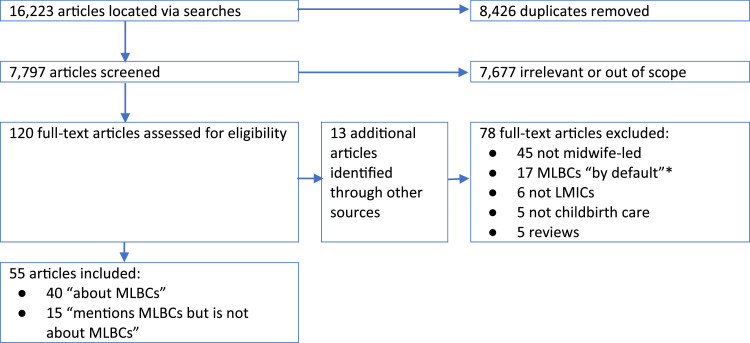
Results of searches for peer-reviewed literature. * The review located several items of literature which described a facility where the midwife was the only available health professional. These were classed as “MLBCs by default” and excluded from the analysis because there was no evidence that this was a deliberate policy based on a clear commitment to the philosophy of midwife-led care.

The final selection process for the peer-reviewed literature is illustrated in Fig. 1. In total, 16,223 references were identified, of which 8426 were duplicates. Of the 7797 remaining references, 7677 did not meet the inclusion criteria, leaving 120 which had a full-text review, plus 13 additional references located *via* hand searches (total = 133). Of these 133, 78 were excluded after full-text review, leaving 55 items included.

The included items fell into two categories: (1) “about MLBCs” (n=40), i.e. the MLBC was the main focus of the item and it provided information relating to both the primary and secondary research questions, and (2) “mentions MLBCs but is not about MLBCs” (n=15), i.e. the item confirmed the existence of one or more MLBCs and therefore contributed to answering the first research question, but not the secondary question. Both types of paper were included in the analysis of the first question, but only the first group of 40 items were included in the analysis for the secondary question.

The 55 included articles covered 14 LMICs. All but one of the articles were in English, with one in Spanish. Analysis of the year of publication showed no obvious pattern over time: 28 were published in the period 2012–2016, and 27 in the period 2017–2022.

Of the 40 articles “about MLBCs”, most (36) were research studies, three were opinion pieces, and one was a conference abstract. The 36 research studies had a variety of aims and methods. The most common aims were to examine the experiences of midwives or clients. Some (n=12) aimed to quantify maternal and neonatal outcomes and/or compare these with other birth settings. In terms of methods, 17 studies used quantitative methods, 16 used qualitative methods, and 3 used mixed methods. Of the 17 quantitative studies, 9 involved retrospective analysis of routine health facility records, 7 involved a survey (5 surveyed staff and 2 surveyed clients), and one was a randomized controlled trial. Of the 16 qualitative studies, 8 interviewed MLBC staff, 5 interviewed MLBC clients, 1 interviewed both staff and clients and 2 were case studies. Just one of the mixed-method studies collected data from both staff and clients.

In total 74 items of grey literature were selected for full text review, of which 46 were included in the analysis. This brought the total number of items of literature to 101.

### In which LMICs do MLBCs exist?

There is evidence from the literature and/or the survey that MLBCs existed in 57 LMICs (Table 3). However, in most cases the evidence was weak, i.e. the country was mentioned just once, either in the literature or the scoping survey. The evidence was stronger for 24 countries, i.e. the country was mentioned in two or three out of: peer-reviewed literature, grey literature and survey response. These 24 countries appear in the top section of Table 2.

**Table 3:** Countries for which there was evidence of MLBCs.

	Country (alphabetical order)	No. of items of peer-reviewed literature (references)	No. of items of grey literature (references)	Survey indicated MLBC(s)
Strong evidence (from two or three of the data source types)	Afghanistan	–	n=2 (Jobs.Af, 2021; Summers, 2021)	✓
	Bangladesh	n=2 (Mahmood et al., 2019; Wallace, 2019)	n=6 (Amin et al., 2020; Begum, 2019; Every Mother Counts, 2022; Goodbirth.net, 2021; Jahan, 2018; Michel-Schuldt et al., 2020a)	✓
	Brazil	n=10 (Caldas Nicacio et al., 2016; da Silva et al., 2013; 2012; Freitas et al., 2019; Nunes et al., 2016; Progianti et al., 2013; Rodrigues Duarte et al., 2019; Santos et al., 2015; Schneck et al., 2012; Viana et al., 2012)	n=2 (Goodbirth.net, 2021; Silva, 2019)	nr
	Ecuador	–	n=2 (Goodbirth.net, 2021; New Life Ecuador, 2020)	✓
	Fiji	–	n=2 (United Nations Population Fund, 2019; Vula, 2018)	✓
	Gambia	–	n=2 (Sheikh Tihami I Nyass Foundation, 2022; Tripadvisor, 2015)	✓
	Ghana	–	n=2 (Goodbirth.net, 2021; United Nations Population Fund, 2021b)	✓
	Guatemala	–	n=2 (Asociación Corazón del Agua, 2021; Goodbirth.net, 2021)	✓
	Guinea	–	n=1 (Goodbirth.net, 2021)	✓
	Haiti	n=1 (Floyd and Brunk, 2016)	n=5 (Goodbirth.net, 2021; MamaBaby Haiti, 2022; Midwives for Haiti, 2015; Second Mile Haiti, 2022; Williams, 2018)	✓
	India	n=2 (Bogren and Erlandsson, 2021; David et al., 2012)	n=4 (Birthvillage, 2022; Goodbirth.net, 2021; The Birth Home, 2020; The Sanctum, 2022)	✓
	Indonesia	n=3 (Diba et al., 2019; Erawati et al., 2020; Zulfa et al., 2021)	n=3 (Bumi Sehat Foundation International, 2018; Reis, 2012; United Nations Population Fund, 2020)	✓
	Iran	n=4 (Moudi et al., 2014; Moudi and Tabatabaei, 2016; Shahinfar et al., 2021; Zolala et al., 2019)	–	✓
	Malawi	–	n=5 (Chatonda, 2021; Chodzaza and Moyo, 2021; Goodbirth.net, 2021; Kondowe, 2019; Seed Global Health, 2020)	✓
	Mexico	n=2 (Alonso et al., 2018; 2021)	–	✓
	Morocco	–	n=3 (Annuaire Gratuit Maroc, 2022; Goodbirth.net, 2021; Le360 (avec MAP), 2018)	✓
	Pakistan	n=3 (Akhtar et al., 2017; Anwar et al., 2014; Shahnaz et al., 2015)	n=3 (Goodbirth.net, 2021; The Indus Hospital, 2021; United Nations Population Fund, 2021a)	✓
	Philippines	n=1 (Wallace, 2019)	n=2 (Goodbirth.net, 2021; Mercy in Action, 2022)	✓
	Sierra Leone	n=1 (Ngongo et al., 2013)	n=2 (Goodbirth.net, 2021; Jones, 2014)	nr
	South Africa	n=22 (Abrahams et al., 2022; 2018; Anonymous, 2012; Dutton and Knight, 2020; Hofmeyr et al., 2014; Horner and Mashamba, 2014; Kennedy et al., 2012; Khoza-Shangase and Harbinson, 2015; Lau et al., 2014; Malatji and Madiba, 2020; Malesela, 2021; Mehta et al., 2018; Oosthuizen et al., 2022; 2020; 2019; Oosthuizen et al., 2017; Pattinson, 2015; Petersen Williams et al., 2014; 2018; Springer et al., 2020; Stellenberg and Ngwekazi, 2016; Zitha and Mokgatle, 2020)	n=2 (Goodbirth.net, 2021; Mother Instinct, 2022)	✓
	Uganda	–	n=3 (Global Force for Healing, 2021; Mother Health International, 2016; We Care Solar, 2013)	✓
	Yemen	–	n=1 (Goodbirth.net, 2021)	✓
	Zambia	–	n=1 (Goodbirth.net, 2021)	✓
	Zimbabwe	–	n=1 (Goodbirth.net, 2021)	✓
Weak evidence (from just one of the three data source types)	Argentina	–	n=1 (Goodbirth.net, 2021)	✕
	Benin	–	–	✓
	Bolivia	–	n=1 (Goodbirth.net, 2021)	✕
	Bulgaria	–	–	✓
	Cambodia	–	n=1 (Goodbirth.net, 2021)	nr
	Chad	–	–	✓
	China	n=2 (Jiang et al., 2018; Wang et al., 2012)	–	nr
	Comoros	–	–	✓
	Congo	–	n=1 (Goodbirth.net, 2021)	nr
	Democratic Republic of the Congo	–	n=1 (Goodbirth.net, 2021)	✕
	Iraq	–	n=1 (Médecins Sans Frontières, 2014)	nr
	Kenya	–	n=1 (Africa Mission Services, 2020)	✕
	Kyrgyzstan	–	–	✓
	Lebanon	–	n=2 (Médecins Sans Frontières, 2019; Medina and Trinh, 2022)	✕
	Liberia	–	–	✓
	Madagascar	–	n=1 (Goodbirth.net, 2021)	✕
	Mali	–	n=1 (Goodbirth.net, 2021)	✕
	Mozambique	–	n=1 (Goodbirth.net, 2021)	nr
	Myanmar	–	n=1 (Goodbirth.net, 2021)	✕
	Nepal	n=2 (Sapkota et al., 2012; Shah, 2016)	–	✕
	Palestine	–	n=1 (Goodbirth.net, 2021)	nr
	Peru	–	n=1 (Goodbirth.net, 2021)	nr
	Romania	–	n=1 (Newsbeezer.com, 2020)	nr
	Rwanda	–	n=1 (Goodbirth.net, 2021)	✕
	Senegal	–	n=1 (Goodbirth.net, 2021)	✕
	Somalia	–	–	✓
	Sri Lanka	–	–	✓
	Syria	–	–	✓
	Thailand	–	n=1 (Goodbirth.net, 2021)	nr
	Turkey	n=1 (Bayoglu Tekin et al., 2015)	–	nr
	Ukraine	–	n=1 (Goodbirth.net, 2021)	nr
	Vanuatu	–	–	✓
	Viet Nam	–	n=2 (Goodbirth.net, 2021; United Nations Population Fund, 2017)	nr

nr = the country did not respond to the survey (or, in the case of Ukraine, was not invited to participate). ✕ = the survey response indicated no MLBCs.

**Fig. 2:**
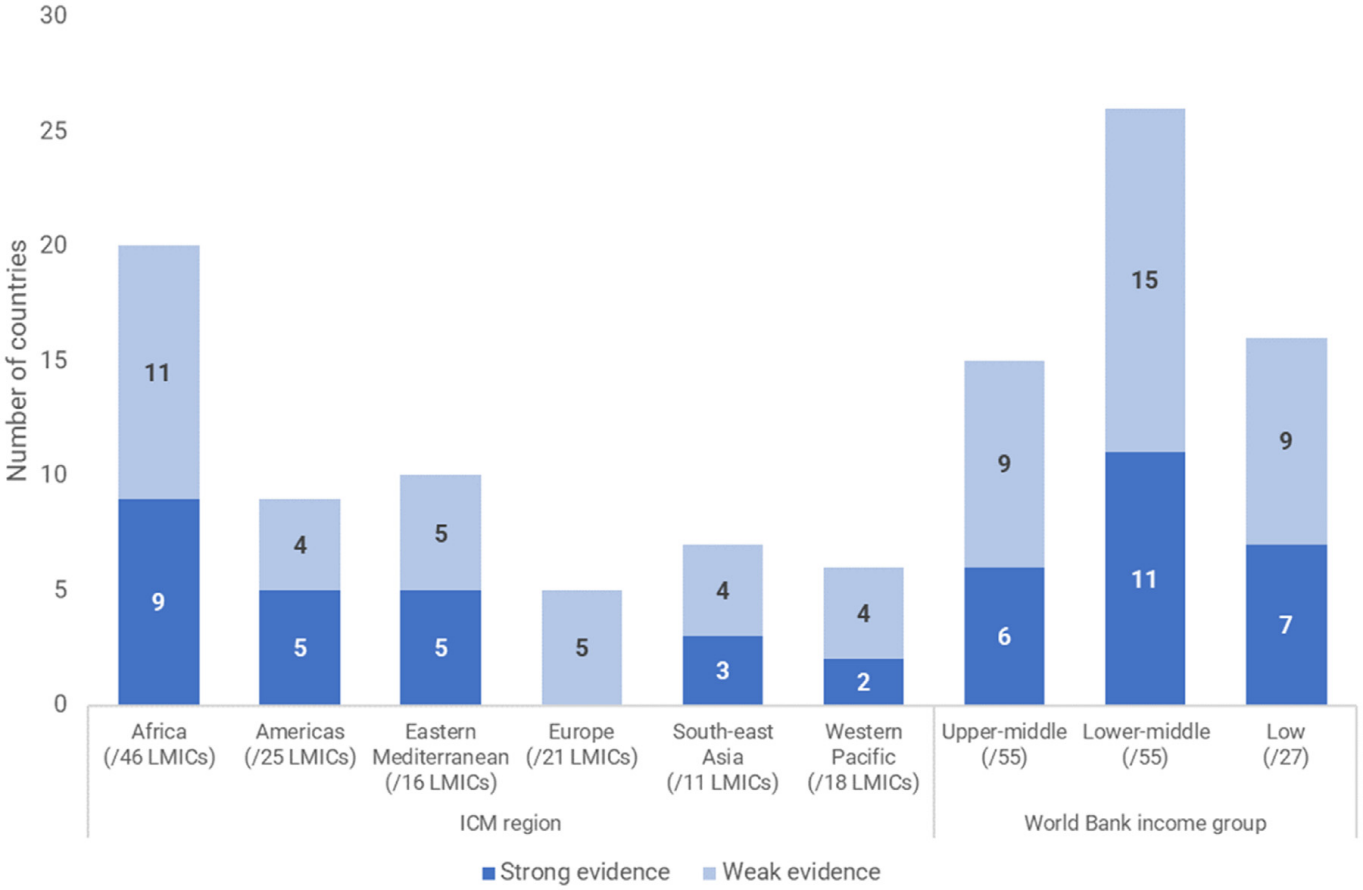
Number of countries with evidence of MLBCs, by ICM region and World Bank income group.

Of the 57 LMICs with evidence indicating the existence of MLBCs, 16 were low-income countries (out of 27 low-income countries in the world), 26 were lower-middle-income (out of 55 in the world), and 15 were upper-middle income (out of 55 in the world) (Fig. 2). Just one of the studies from the peer-reviewed literature was from a low-income country – the other 15 low-income countries with MLBCs were identified *via* the grey literature and scoping survey. Two upper-middle-income countries - Brazil and South Africa – accounted for more than half of the located peer-reviewed literature. Fig. 2 also shows that in every ICM region there were at least five LMICs with evidence of MLBCs. Europe was the only region with no strong evidence of MLBCs in its 21 LMICs.

In the scoping survey, eight countries reported that the country had no MLBCs at present, but there were plans to establish them: Argentina, Egypt, Lesotho, Lebanon, Madagascar, Nepal, Solomon Islands, and South Sudan.

Of the 32 countries who indicated in the scoping survey that they had MLBCs, just over half (n=17) said there were more than 10 MLBCs in the country, 5 said there were 3–10 MLBCs, and 6 said there were just one or two MLBCs. The remaining 4 countries did not know or did not state how many MLBCs existed.

### Main characteristics of MLBCs in LMICs

We found 40 different names in use to describe MLBCs. The most commonly used name was “birth(ing) centre” (17 countries), followed by “birth(ing) house/home” (10 countries). The name ‘MLBC’ was only reported once (in Pakistan's survey response, which may have been influenced by the use of this term in the questionnaire). Eighteen of the names included the word “midwife” or “midwifery”, and 11 included the word “birth”. A full list of names and the country/ies in which each was used can be found in Table S.3 in the supplementary file.

Many countries used more than one name. This may be due to different names being used for different types of MLBCs (e.g. Afghanistan, India, Malawi, Pakistan, South Africa). It may also be due to different authors using different terminology, either because no standard name is in use or because the name can be translated into English in different ways (e.g. in Brazil, Indonesia, Philippines). Multiple names within a country also seems to be an effect of MLBCs not being well integrated within the health system (e.g. Gambia, Haiti, Iran).

The remaining Findings are limited to the 24 countries with strong evidence of MLBCs, unless otherwise specified.

By far the most common type of MLBC was freestanding (located on a separate site from a hospital). This type of MLBC was identified in all 24 countries. The next most common type was onsite MLBCs, i.e. located within a hospital obstetric unit (13 countries), then alongside, i.e. on the same site as a hospital obstetric unit but not within it (8 countries). Table 4 shows that more than one type was identified in most countries with MLBCs, but in nine countries only freestanding MLBCs were identified. In the survey and grey literature, a few countries (e.g. Afghanistan, Indonesia, Malawi) specified that their freestanding MLBCs included services provided at the midwife's own home.

**Table 4:** Types of MLBC identified in each country.

Country	Freestanding	Onsite	Alongside	No. of types identified
Afghanistan	✓	✓	✓	3
Bangladesh	✓	✓		2
Brazil	✓	✓	✓	3
Ecuador	✓			1
Fiji	✓	✓		2
Gambia	✓			1
Ghana	✓	✓	✓	3
Guatemala	✓			1
Guinea	✓	✓	✓	3
Haiti	✓			1
India	✓	✓		2
Indonesia	✓	✓	✓	3
Iran	✓		✓	2
Malawi	✓	✓		2
Mexico	✓	✓		2
Morocco	✓			1
Pakistan	✓	✓		2
Philippines	✓		✓	2
Sierra Leone	✓			1
South Africa	✓	✓	✓	3
Uganda	✓			1
Yemen	✓			1
Zambia	✓	✓		2
Zimbabwe	✓			1
Number of countries	24/24	13/24	8/24	

There was a fairly even mix in terms of the sector in which MLBCs operate. In over half of the 24 countries there were some public- and some private-sector MLBCs. Three countries only had public-sector MLBCs: Brazil, Fiji, and Iran. Seven countries had only private-sector MLBCs: Afghanistan, Guatemala, Guinea, Haiti, Sierra Leone, Yemen, and Zimbabwe.

**Fig. 3:**
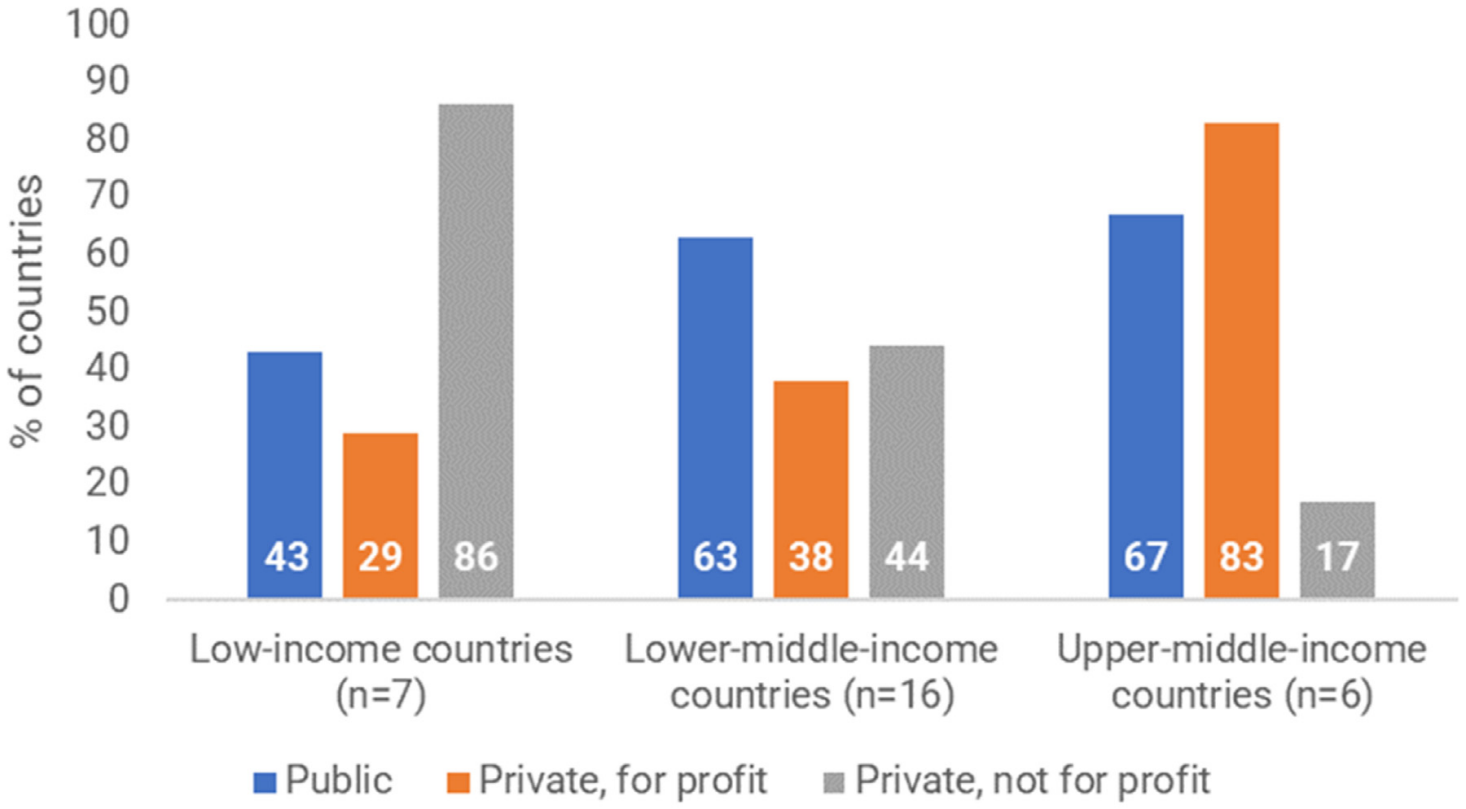
Sector(s) in which MLBCs exist in 29 countries which provided survey data about their MLBCs. Note: in this analysis we have included all countries who stated in the survey that they had MLBCs, including those that did not feature in the literature.

The survey results indicated a strong relationship between country income group and sector (Fig. 3). Low-income countries were much more dependent on the private, not-for-profit sector (e.g. non-governmental or faith-based organisations), whereas public-sector MLBCs were more common in middle-income countries. Private for-profit MLBCs were much more common in upper-middle-income countries than in low- and lower-middle-income countries.

In most of the 24 countries there was evidence from at least one source that the country had MLBCs in both urban and rural areas. Conversely, in Brazil and Malawi MLBCs were identified only in urban areas, and in Morocco only in rural areas. The urban-rural classification was based on the definition used by the author (if an item of literature) or by the survey respondent (if a survey response).

Amongst the 24 countries with strong evidence, most had a mix of staffing models, i.e. within a country some MLBCs were staffed by midwives and some by a multidisciplinary team comprising midwives and other health workers. The exceptions were Bangladesh and Yemen, where there was no evidence of MLBCs staffed by multidisciplinary teams. Twelve countries indicated in the survey that, in at least some of their MLBCs, care was provided by a single midwife working alone: Afghanistan, Benin, Comoros, Ecuador, Ghana, Guatemala, Indonesia, Mexico, Uganda, Vanuatu, Yemen, and Zambia. Six countries specified that caseload midwifery was practised at some or all of their MLBCs: Fiji, Ghana, Guatemala, Mexico, Vanuatu, and Zambia.

In the peer-reviewed literature, barriers were identified to the provision of the midwifery philosophy of care in MLBCs. For example, many Brazilian and Indian midwives had previously trained and worked in hospital obstetric units and found it difficult to provide care in another way (da Silva et al., 2012; David et al., 2012). In South Africa, alignment with the midwifery philosophy was found to be dependent on the motivation of individual midwives (Dutton and Knight, 2020). In general, motivation was low, and disrespect and abuse were common (Dutton and Knight, 2020; Malatji and Madiba, 2020; Oosthuizen et al., 2020). On the other hand, the literature from Mexico and Pakistan described how, in at least some parts of the country, birth was viewed as a family/community event. The care provided at MLBCs fitted well with this culture, e.g. by encouraging partners or other community members to accompany women through labour and birth

Similarly, in some countries MLBC clients were reported as being unused to the midwifery philosophy of care. For example, some of the literature from Brazil noted that many clients expected to take a passive role. Efforts to encourage their active participation were unsuccessful, especially if they had been allocated to MLBC care simply because the MLBC was close to their home. If they wanted or expected to give birth under a medical model they could feel as though the care had not met their expectations (Nunes et al., 2016).

Furthermore, when the MLBC was onsite, the proximity and accessibility of doctors meant that midwives (generally lower in the professional hierarchy) did not feel empowered to overrule doctors when the midwifery model and the medical model were at odds (Nunes et al., 2016; Rodrigues Duarte et al., 2019). In countries where the medical model of care was dominant, this problem was exacerbated because service users tended to trust doctors more than midwives even in an MLBC where the midwives were the lead professionals (Anwar et al., 2014).

In addition to childbirth services, MLBCs in most of the 24 countries also offered antenatal care and/or postnatal care. However, family planning services were rarely mentioned in the literature except in: Bangladesh, Gambia, Guatemala, Haiti, and Sierra Leone. It is not possible to tell in which countries MLBCs offered the full continuum of care, because different individual MLBCs featured in the literature and we cannot assume that within a country all MLBCs offered the same range of services.

One of the main defining features of the MLBCs featured in the peer-reviewed literature was that they specialized in care for uncomplicated births. However, this was not always achieved in practice. For example, one study from South Africa found that 20% of MLBC clients were classified as ‘high risk’ at booking, of whom 21% gave birth in the MLBC (Homer et al., 2019). In most countries MLBCs routinely referred to higher levels of care in an emergency. However, in some settings (e.g. Indonesia and South Africa), the referral system did not always work well (e.g. difficulties in obtaining informed consent, complicated administrative processes, transport problems), which meant that access to emergency care could not always be ensured (Diba et al., 2019; Dutton and Knight, 2020; Erawati et al., 2020; Homer et al., 2019). In Mexico, there were no established communication channels between the MLBCs and the referral hospitals, leading to delayed transfers when needed, and to clients being “scolded” at the hospital for attempting an MLBC birth (Alonso et al., 2021). By contrast, in Bangladesh the MLBCs that were operated by a nongovernmental organization (NGO) had a memorandum of understanding with (and administrative staff deployed within) higher-level facilities, which facilitated emergency referrals (Wallace, 2019).

Information about costs and payment mechanisms was scarce in both the peer-reviewed literature and the survey responses. Where information was provided, it indicated more than one payment mechanism existed in most countries, i.e. different MLBCs had different arrangements. In their survey responses, four countries indicated that all MLBCs were fully funded by the state: Brazil, Fiji, India, and South Africa (however, the grey literature indicated that some MLBCs were financed by user fees). Similarly, Uganda was the only country to indicate in the survey that user fees were the only payment mechanism, although the grey literature suggests that donor/NGO funds are also used in Uganda. Four countries appeared to rely solely on donor/NGO funding for MLBCs: Afghanistan, Bangladesh, Haiti, and Sierra Leone.

Papers from Mexico (Alonso et al., 2021) and Pakistan (Akhtar et al., 2017), described a sliding cost scale, with lower user fees for poorer clients, subsidized through donor funds. In Pakistan, this was contrasted with “fixed” hospital fees. None of the literature made a valid comparison of the cost of MLBC birth against the cost of other birth settings. There was no discussion of the impact of additional expenses for transport, medicines, supplies and medical tests, nor the costs of establishing and running an MLBC.

Some of the peer-reviewed literature included an attempt to measure the impact of MLBCs, using a variety of outcome measures. Most reported positive outcomes to MLBC births such as low mortality and morbidity rates (Anonymous, 2012; David et al., 2012; Moudi and Tabatabaei, 2016; Ngongo et al., 2013; Progianti et al., 2013; Schneck et al., 2012), low intervention rates (Alonso et al., 2021; Caldas Nicacio et al., 2016; David et al., 2012; Ngongo et al., 2013; Schneck et al., 2012; Wallace, 2019) and high quality of care (Akhtar et al., 2017; da Silva et al., 2013; David et al., 2012; Freitas et al., 2019). The main exceptions were the evidence from South Africa of high rates of disrespect and abuse (Dutton and Knight, 2020; Malatji and Madiba, 2020; Oosthuizen et al., 2020, 2017; Zitha and Mokgatle, 2020), and the evidence from Brazil of some ineffective or potentially harmful practices in MLBCs (da Silva et al., 2013; Freitas et al., 2019).

To accurately measure the impact of MLBCs, a comparator is needed so that outcomes at MLBCs can be compared with outcomes at other birth settings. Not all of the studies in this review had the aim of comparing MLBCs with other birthplace options. Amongst those that did, the most common comparator was the obstetric unit of a public sector hospital (Alonso et al., 2021; Caldas Nicacio et al., 2016; Diba et al., 2019; Freitas et al., 2019; Hofmeyr et al., 2014; Moudi et al., 2014; Moudi and Tabatabaei, 2016; Schneck et al., 2012). Other studies compared MLBC outcomes with national figures (da Silva et al., 2012), or compared the population served by an MLBC against a ‘control’ population without access to an MLBC (Wallace, 2019). Of those studies that did make comparisons, sometimes the comparisons were not fully valid, e.g. they excluded the outcomes of MLBC clients who transferred to hospital care, or they did not take into account the fact that MLBC clients usually had a lower risk profile than the comparator population.

## Discussion

This study found strong evidence that MLBCs exist in 24 LMICs, and weaker evidence that they exist in many more. Only a minority of these countries feature in the peer-reviewed literature, so an analysis based solely on the peer-reviewed literature may present a skewed picture which cannot be generalized to a much wider range of countries. In particular, most of the peer-reviewed literature comes from middle-income countries such as Brazil and South Africa, where there is a network of public-sector MLBCs well established within the national health system. The grey literature and the survey indicated that MLBCs exist in a much broader range of settings, where different opportunities and challenges may exist.

The MLBCs we identified did not all adhere fully to ICM's working definition of an MLBC. Although they all specialised in uncomplicated pregnancies and identified midwives as the lead professionals providing care, other elements of the definition occurred in some places but not in others. For example, there can be barriers to operating fully within the midwifery philosophy of care, ensuring access to emergency care when needed, and encouraging the participation of clients in decisions about their care. This indicates that the working definition may need to be reviewed and perhaps revised to make it applicable in a wider range of contexts.

Many of our findings echo those from research in HICs, e.g. professional hierarchies and rivalries can be problematic (Behruzi et al., 2017; McCourt et al., 2014). However, this study indicates that some issues may be specific to all or some LMICs. For example, MLBC clients in HICs tend to be relatively wealthy (Brocklehurst et al., 2011), whereas in many LMICs, MLBCs serve mostly clients from poor and marginalized communities. It is notable that in low-income countries MLBCs were mostly provided by private, not-for-profit sources, which means access to care for clients from poor and marginalized communities is reliant on their ongoing support. Research from HICs indicates that outcomes for low-risk pregnancies tend to be slightly better in freestanding MLBCs than in onsite MLBCs (Brocklehurst et al., 2011), whereas this study highlights that weak referral systems can adversely affect birth outcomes in freestanding MLBCs. This is of particular concern because this study found that freestanding MLBCs were the most common type in LMICs.

The findings echo related research from LMICs, e.g. recent studies in Bangladesh and India concluded that the successful introduction of MLBCs is to some extent dependent on: midwives being enabled to operate to their full scope of practice in line with the midwifery philosophy of care *via* education and training which is aligned with this philosophy, demand creation activities amongst potential clients, and professional support for midwifery leadership (Bogren et al., 2022; Pappu et al., 2023). This indicates that – for MLBCs to be successful in improving maternal and neonatal outcomes in LMICs - in some contexts there is a need for more efficient legislation that supports midwives to practise autonomously within their full scope, to improve understanding about the midwifery philosophy of care and to strengthen referral systems.

This study located very little evidence about costs and payment mechanisms for MLBCs in LMICs, which acts as a barrier for determining ongoing financial viability or further investment for private or public providers of MLBCs. However, a lack of economic evidence is also an issue in research about maternity care in high-income countries. Similarly, the evidence is scant and poor quality in relation to how maternal and newborn health outcomes amongst MLBC clients in LMICs compare with other available birth settings. These are important knowledge gaps which future research should aim to fill.

This study is subject to a number of limitations. Some of the literature was published up to ten years ago, so it may not be reflective of current practices in the featured countries, e.g. the two peer-reviewed papers from China related to a trial of the MLBC model, and the lack of more recent publications on this topic implies that this model of care was never scaled and therefore may no longer exist. Some of the literature (in particular the grey literature) did not make clear their definition of a midwife and an MLBC, so it is possible that we included some literature about facilities that are not MLBCs. In particular, we made no attempt to establish whether the midwives mentioned in the literature adhered to the ICM definition of a midwife (International Confederation of Midwives, 2017a). The exclusion of literature in languages other than English, French and Spanish means that we may have excluded some countries which have MLBCs but have not yielded publications in one of these three languages. Finally, the large number of names used to describe MLBCs implies that, had we used a wider range of search terms, we may have located literature about MLBCs from a larger number of countries.

## Conclusions

This study provides up-to-date evidence about which countries have MLBCs, and some information about the characteristics of MLBCs in LMICs. Low- and lower-middle-income countries were more likely than upper-middle-income countries to have MLBCs. The most common type of MLBC was freestanding. Public-sector midwife-led birthing centres were more common in middle-income than in low-income countries. Some were staffed entirely by midwives and some by a multidisciplinary team. We identified challenges to the midwifery philosophy of care, legislative and regulatory requirements, and effective referral systems.

The peer-reviewed literature does not provide a comprehensive picture of the locations and characteristics of MLBCs in LMICs. Many of our findings echo those from high-income countries, but some appear to be specific to some or all LMICs. The study highlights knowledge gaps, including a lack of evidence about the impact and costs of MLBCs in LMICs.

## Ethical approval

Not applicable

## CRediT authorship contribution statement

**Andrea Nove:** Conceptualization, Data curation, Methodology, Resources, Formal analysis, Writing – original draft, Supervision. **Oliva Bazirete:** Data curation, Methodology, Resources, Supervision. **Kirsty Hughes:** Data curation, Methodology, Resources, Supervision. **Sabera Turkmani:** Data curation, Methodology, Resources, Supervision. **Emily Callander:** Supervision. **Vanessa Scarf:** Supervision. **Mandy Forrester:** Conceptualization, Supervision. **Shree Mandke:** Conceptualization, Supervision. **Sally Pairman:** Conceptualization, Supervision. **Caroline SE Homer:** Conceptualization, Data curation, Methodology, Resources, Supervision.

## Declaration of Competing Interest

None declared.

## Appendix Supplementary materials

Image, application 1
