# Supplementary material for: Which low- and middle-income countries have midwife-led birthing centres and what are the main characteristics of these centres? A scoping review and scoping survey
Source: Midwifery. 2023 Aug;123:103717. doi: 10.1016/j.midw.2023.103717 (PMC10281083; doi:10.1016/j.midw.2023.103717)
Supplement: Supplementary file 1 [file mmc1.docx]

**Supplementary file**

Table S.1 gives details of the databases and search terms used in the scoping review of the peer-reviewed literature.

**Table S.1: Databases and search terms for peer-reviewed literature**

| **Database** | **Search terms** |
| --- | --- |
| CINAHL | AB (midwi* OR sage-femme OR matrona or partera or "enfermera obstétrica") AND AB (unit* OR cent* OR unidad OR "maison de naissance" OR "casa de parto" OR “casa de partos”)  Filters: human; language = English, French or Spanish; publication year 2012-2022; geography = Europe, Continental Europe, Africa, Asia, Middle East, Mexico & central/south America |
| Cochrane Library | Title Abstract Keyword: (midwi* OR sage-femme OR matrona OR partera OR “enfermera obstétrica”) AND (unit* OR cent* OR unidad OR “maison de naissance” OR “casa de parto” OR “casa de partos”)  Filter: publication year 2012-2022; Cochrane reviews |
| EMBASE | ('enfermera obstétrica':ti,ab,kw OR midwi*:ti,ab,kw OR 'sage femme':ti,ab,kw OR matrona:ti,ab,kw OR partera:ti,ab,kw) AND (unit*:ti,ab,kw OR cent*:ti,ab,kw OR unidad:ti,ab,kw OR 'maison de naissance':ti,ab,kw OR 'casa de parto':ti,ab,kw OR 'casa de partos':ti,ab,kw) AND [2012-2022]/py AND ([english]/lim OR [french]/lim OR [spanish]/lim) AND [humans]/lim |
| LILACS | Title, abstract, subject: (midwi* OR sage-femme OR matrona OR partera OR “enfermera obstétrica”) AND (unit* OR cent* OR unidad OR "casa de parto" OR “casa de partos”)  Filters: language English, French or Spanish; publication year 2012-2022 |
| MEDLINE | AB (midwi* OR sage-femme OR matrona or partera or "enfermera obstétrica") AND AB (unit* or cent* or unidad or "maison de naissance" or "casa de parto" or “casa de partos”)  Filters: human; language = English, French or Spanish; year of publication 2012-2022 |
| PubMed | (midwi*[Title/Abstract] OR sage-femme[Title/Abstract] OR matrona[Title/Abstract] OR partera[Title/Abstract] OR "enfermera obstétrica"[Title/Abstract]) AND (unit*[Title/Abstract] OR cent*[Title/Abstract] OR unidad[Title/Abstract] OR "maison de naissance"[Title/Abstract] OR "casa de parto"[Title/Abstract] OR “casa de partos”[Title/Abstract])  Filters: last 10 years; Humans, English/French/Spanish |
| Sabinet | Abstract: (midwife OR sage-femme) AND (unit OR center OR centre OR "maison de naissance")  Filters: 2012-2022 |
| Scopus | TITLE-ABS-KEY ((midwi* OR sage-femme OR matrona OR partera OR "enfermera obstétrica") AND (unit* OR cent* OR unidad OR "maison de naissance" OR  casa de parto" OR “casa de partos”)) AND PUBYEAR > 2011 AND(LIMIT-TO ( LANGUAGE , "English") OR LIMIT-TO (LANGUAGE, "French") OR LIMIT-TO (LANGUAGE, "Spanish"))  Filters: Geography - exclude individual high-income countries |
| Web of Science | (TS=(midwi* OR sage-femme OR matrona or partera or "enfermera obstétrica")) AND TS=(unit* or cent* or "maison de naissance" or "casa de parto")  Filters: language = English, French or Spanish, Geography = exclude individual high-income countries; Year of publication after 2011 |

Table S.2 details which of the world’s 137 LMICs were invited to contribute to the scoping survey, which organization issued the invitation, and whether or not a completed questionnaire was received from that country.

**Table S.2: Scoping survey response from each country**

|  | **Invited by ICM** | **Invited by UNFPA head office** | **Not invited** |
| --- | --- | --- | --- |
| **Responded** | Afghanistan, Algeria, Argentina, Bangladesh, Benin, Bolivia, Bulgaria, Burkina Faso, Burundi, Chad, Comoros, Costa Rica, Côte d’Ivoire, DRC, Ecuador, Ethiopia, Fiji, Gabon, Gambia, Ghana, Guatemala, Guinea, Guinea-Bissau, Guyana, Haiti, India, Indonesia, Iran, Kenya, Kyrgyzstan, Lebanon, Lesotho, Liberia, Madagascar, Malawi, Mali, Mexico, Mongolia, Morocco, Myanmar, Namibia, Nepal, Nigeria, North Macedonia, Pakistan, Philippines, Rwanda, Senegal, Somalia*, South Africa, South Sudan, Sri Lanka, Tunisia, Uganda, Yemen, Zambia, Zimbabwe.  **N=57** | Angola, Bhutan, Cabo Verde, Colombia, Cuba, Djibouti, Dominica, Dominican Republic, Egypt, El Salvador, Honduras, Jordan, Libya, Maldives, São Tome and Principe, Solomon Islands, Sudan, Syria, Uzbekistan, Vanuatu.         **n=20** | - |
| **Did not respond** | Botswana, Brazil, Cambodia, Cameroon, CAR, Congo, Eswatini, Iraq, Kosovo, Lao PDR, Mauritania, Mozambique, Niger, Palestine, PNG, Paraguay, Peru, Romania, Sierra Leone, Suriname, Tajikistan, Tanzania, Timor-Leste, Togo, Turkey, Viet Nam.  **n=26** | Albania, Armenia, Azerbaijan, Belarus, Belize, Bosnia & Herzegovina, China, DPR Korea, Equatorial Guinea, Eritrea, FSM, Georgia, Grenada, Jamaica, Kazakhstan, Kiribati, Malaysia, Marshall Islands, Mauritius, Moldova, Montenegro, Nicaragua, Panama, St Lucia, St Vincent & the Grenadines, Samoa, Serbia, Thailand, Tonga, Turkmenistan, Tuvalu.  **N=31** | American Samoa, Russia, Ukraine        **n=3** |

* Response related only to Somaliland, and not to the rest of the country.

Table S.3 lists all the names for MLBCs that were found in the literature and/or survey responses.

**Table S.3: Names used for MLBCs**^[[1]](#footnote-1)^

| **Name** | **Country/ies where this name is used** |
| --- | --- |
| Birth(ing) centre (incl. *centro de parto*) | Bangladesh; Brazil; Cambodia; Ghana; Guatemala; Haiti; Iran; Kenya; Lebanon; Mexico; Nepal; Pakistan; Philippines; Romania; Rwanda; Sierra Leone; South Africa |
| Birthing clinic | Philippines |
| Birth(ing) house/home (incl. casa de parto, casa de nacimiento, maison de naissance, casa de parteria, maison d’accouchement) | Brazil; Comoros; Guatemala; Haiti; Mexico; Morocco; Peru; Philippines; South Africa; Uganda |
| Birth(ing) unit | Fiji; South Africa |
| Centre revitalizé | Chad |
| Childbirth centre | Indonesia |
| Community clinic | Gambia |
| Domiciliary clinic | Uganda |
| Independent midwife practice | Indonesia |
| Maternity centre | Haiti; Madagascar; Uganda |
| Maternity clinic | Gambia; Guinea ; South Africa; Zimbabwe |
| Maternity home (incl. *casa materna*) | Brazil; Ghana; Guatemala; Uganda; Viet Nam; Zimbabwe |
| Maternity hospital | Ecuador; Zimbabwe |
| Maternity unit | Malawi; Liberia |
| Midwife centre | Indonesia |
| Midwife(ry) clinic | Philippines; Yemen |
| Midwife-led birth unit | South Africa |
| Midwife-led birthing centre | Pakistan |
| Midwife(ry)-led care centre | Bangladesh |
| Midwife(ry)-led care unit | Bangladesh; Brazil; China; India; Pakistan |
| Midwife-led labour ward | Zambia |
| Midwife(ry)-led unit | Afghanistan; Fiji; Turkey |
| Midwifery-led maternity care centre | Afghanistan |
| Midwife(ry)-led maternity unit | Sierra Leone |
| Midwifery-led ward | Malawi |
| Midwife obstetric unit | South Africa |
| Midwife-run labour room | India |
| Midwifery centre | India; Mexico |
| Midwife private practice unit | Indonesia |
| Midwifery unit | Brazil; Bulgaria, Fiji; Indonesia; Pakistan |
| Mother and child health centre | Somalia |
| Natural birth(ing) centre (incl. *centro de parto natural*) | Brazil; Ecuador; India |
| Normal birth centre (incl. *centro de parto normal*) | Brazil; China; Iran; Syria |
| Normal birth clinic (incl. clinique d’accouchement eutocique) | Benin |
| Normal birth unit | Sri Lanka; Syria; Vanuatu |
| Private midwife(ry) clinic | Indonesia; Iran; Pakistan |
| Safe delivery post | Iran |
| Village midwife practice | Indonesia |
| Women’s centre | Sierra Leone |
| Women’s clinic | Malawi |
| Not stated | Argentina, Bolivia, Congo, DRC, Mali, Mozambique, Myanmar, Palestine, Senegal, Thailand, Ukraine |

**MLBCs scoping survey questionnaire**

Q1a. **Name of respondent**___________________________________________________________

Q1b. **Your email address**_____________________________________________________________

Q2. **Name of midwives’ association** (if you are not representing a midwives’ association, please enter the name of the organization you are representing, e.g. UNFPA) ______________________

Q3. **Country name**________________________________________________________________

Q4. **In your country, are there any midwife-led birthing centres (MLBCs)? A midwife-led birthing centre is a location offering childbirth care in which midwives (or nurse-midwives) take primary professional responsibility for care. It may be located within, beside, or separate from a hospital/health facility maternity unit**.
SELECT ONE ONLY. IF MORE THAN ONE APPLIES, PLEASE SELECT THE ONE NEAREST THE TOP OF THE LIST.

- We have one or more MLBCs– GO TO Q6
- We have one or more midwife-led centres or units, but none of them provide childbirth care - END
- We have one or more health facilities where midwives (or nurse-midwives) are the only available childbirth care provider, but these are not MLBCs - END
- We have no MLBCs but there are plans to establish one or more of them - END
- We used to have one or more MLBCs, but currently we have none – END
- We never had any MLBCs and there are no plans to establish them - END
- Don’t know – ANSWER Q5, THEN END

Q5. **Who else could tell us about MLBCs in your country? If there is someone, please enter their name, organizational affiliation and contact details (e.g. email address, phone number).**

________________________________________________________________________________

Q6. **What is the name used in your country to refer to midwife-led birthing centres? For example, “midwifery unit”, “midwife-led unit”, “midwife obstetric unit”, “normal birth unit”, ”maison de naissance”, “casa de parto” etc. If more than one name is used, please enter all the names.**
PLEASE ANSWER IN YOUR OWN WORDS

________________________________________________________________________________

Q7. **How many midwife-led birthing centres currently exist in your country?**SELECT ONE ONLY. AN ESTIMATE IS ACCEPTABLE IF YOU DO NOT KNOW THE EXACT NUMBER.

- 1 or 2
- 3-5
- 6-10
- More than 10
- Don’t know

Q8. **What type(s) of midwife-led birthing centres currently exist in your country?**
SELECT ALL THAT APPLY

- ‘On-site’ (located within the obstetric unit of a hospital)
- ‘Alongside’ (on the same site as a hospital obstetric unit, but not within the obstetric unit)
- ‘Freestanding’ (located on a separate site, away from a hospital obstetric unit)
- Other (please specify)
- Don’t know

Q9. **In which location(s) do midwife-led birthing centres exist in your country?**
SELECT ALL THAT APPLY

- The capital city
- Other urban location(s)
- Village(s) or rural location(s)
- Other (please specify)
- Don’t know

Q10. **Who operates the midwife-led birthing centres in your country?**SELECT ALL THAT APPLY

- Public sector
- Private sector, for profit
- Private sector, not-for-profit (e.g. NGO, faith-based organisation, professional association)
- Other (please specify)
- Don’t know

Q11. **What services are provided in the midwife-led birthing centres (MLBCs) in your country?**
SELECT ONE ONLY

- All MLBCs provide childbirth care only, and no other services
- All MLBCs provide childbirth care plus additional services such as antenatal care, postnatal care, family planning
- Some MLBCs provide only childbirth care, and some provide childbirth care plus additional services
- Other (please specify)
- Don’t know

Q12. **Is the childbirth care in the midwife-led birthing centres in your country provided by…**
SELECT ONE ONLY

- … midwives (or nurse-midwives) only,
- midwives (or nurse-midwives) and other professionals, e.g. doctors and nurses,
- midwives (or nurse-midwives) and other health workers, e.g. auxiliary health workers, community health workers
- it varies – they all have different staffing models,
- other (please specify)
- Don’t know

Q13. **What models of care are available in midwife-led birthing centres in your country?**SELECT ALL THAT APPLY

- Care provided by a single midwife (or nurse-midwife) working alone
- Care provided by a small team of midwives or nurse-midwives
- Caseload model (where a small group of midwives/nurse-midwives provide continuity of antenatal, childbirth and postnatal care to a defined number of women)
- Care provided by a multi-disciplinary team, led by midwives or nurse-midwives
- Other (please specify)
- Don’t know

Q14. **How are the midwife-led birthing centres in your country funded?**SELECT ALL THAT APPLY

- User fees (paid directly by the women/families who access care)
- Health insurance schemes
- Public funds (from the government)
- Donor/charity/private sector funds
- Other (please specify)
- Don’t know

Q15i. **If someone wanted to study the impact and effectiveness of midwife-led birthing centres (MLBCs) in your country, how easy or difficult would it be to access the following types of data and evidence?**
SELECT ONE ANSWER FOR EACH TYPE OF DATA/EVIDENCE

|  | **Easy** | **Possible,  but not easy** | **Impossible** | **Don’t know** |
| --- | --- | --- | --- | --- |
| a. A list of all the MLBCs in the country and their locations |  |  |  |  |
| b. Number of births per month in MLBCs |  |  |  |  |
| c. Range of services offered at MLBCs |  |  |  |  |
| d. Staffing numbers in MLBCs |  |  |  |  |
| e. Data about the midwives/nurse-midwives working in MLBCs, e.g. their age, gender, ethnic group, qualifications, year of first registration as a midwife |  |  |  |  |
| f. Data on availability of essential drugs and equipment at MLBCs, e.g. drug stockout rates |  |  |  |  |

Q15ii. **And how easy or difficult would it be to access the following types of data and evidence?**
SELECT ONE ANSWER FOR EACH TYPE OF DATA/EVIDENCE

|  | **Easy** | **Possible,  but not easy** | **Impossible** | **Don’t know** |
| --- | --- | --- | --- | --- |
| g. Number of mothers transferred from MLBCs to hospital when complications arise |  |  |  |  |
| h. Number of newborns transferred from MLBCs to hospital when complications arise |  |  |  |  |
| i. Number of maternal deaths among MLBC clients |  |  |  |  |
| j. Number of neonatal deaths among MLBC clients |  |  |  |  |
| k. Number of stillbirths among MLBC clients |  |  |  |  |
| l. Number of MLBC clients (women) admitted to intensive care |  |  |  |  |
| m. Other measures of maternal morbidity, e.g. postpartum haemorrhage rate, perineal trauma |  |  |  |  |

Q15iii. **And how easy or difficult would it be to access the following types of data and evidence?**
SELECT ONE ANSWER FOR EACH TYPE OF DATA/EVIDENCE

|  | **Easy** | **Possible,  but not easy** | **Impossible** | **Don’t know** |
| --- | --- | --- | --- | --- |
| n. Number of newborns of MLBC clients admitted to neonatal intensive care or high-dependency unit |  |  |  |  |
| o. Other measures of newborn morbidity among MLBC clients, e.g. Apgar score, number needing resuscitation |  |  |  |  |
| p. The % of MLBC clients giving birth vaginally, by c-section, and by instrumental birth |  |  |  |  |
| q. Measures of women’s satisfaction with the care provided in MLBCs |  |  |  |  |
| r. Measures of the job satisfaction of midwives working in MLBCs |  |  |  |  |
| s. Cost of establishing an MLBC |  |  |  |  |
| t. Monthly or annual running costs for an MLBC |  |  |  |  |
| u. How the costs for MLBCs compare with the costs of providing other models of care |  |  |  |  |

Q16. **Is there any other information you would like to share about MLBCs in your country?**
PLEASE ANSWER IN YOUR OWN WORDS

______________________________________________________________________________

THANK YOU FOR YOUR HELP!

1. We cannot categorically state that the names listed in the table are in regular use in the country, but they have been used when writing about the country and/or in the survey response. [↑](#footnote-ref-1)
